# Prevalence and determinants of low social support during pregnancy among Australian women: a community-based cross-sectional study

**DOI:** 10.1186/s12978-021-01210-y

**Published:** 2021-07-27

**Authors:** Asres Bedaso, Jon Adams, Wenbo Peng, David Sibbritt

**Affiliations:** 1 College of Medicine and Health Sciences, Faculty of Health, School of Nursing, Hawassa University, Hawassa, Ethiopia; 2 Australian Centre for Public and Population Health Research, School of Public Health, Faculty of Health, University of Technology Sydney, Ultimo, NSW Australia

**Keywords:** Low social support, Emotional support, Affectionate support, Tangible support, Mental health, Women, Pregnancy

## Abstract

**Background:**

Pregnancy is a time for women in which the need for social support is crucial. Social support reduces stressors and improves the emotional and physical well-being of pregnant women. Women receiving low social support during pregnancy are at risk of substances use, developing mental illness, and adverse birth outcomes. The current study aims to determine the prevalence and determinants of low social support during pregnancy among Australian women.

**Methods:**

Data were obtained from the 1973–1978 cohort of Australian Longitudinal Study on Women’s Health (ALSWH) and those who report being pregnant (n = 493) were included in the current analyses. Social support was assessed using Medical Outcomes Study Social Support index (MOSS). A logistic regression model was applied to identify determinants of low social support, separately for each MOSS domain.

**Result:**

The study found that 7.1% (n = 35) of pregnant women reported low social support. Significant determinants of low emotional support were non-partnered (AOR = 4.4, 95% CI: 1.27, 14.99), difficulty managing on available income (AOR = 3.1, 95% CI: 1.18, 8.32), experiencing depressive symptoms (AOR = 8.5, 95% CI: 3.29, 22.27) and anxiety symptoms (AOR = 2.9, 95% CI: 1.26, 7.03). Significant determinants of low affectionate support were suffering from depressive symptoms (AOR = 5.3, 95% CI: 1.59, 17.99), having anxiety symptoms (AOR: 6.9, 95% CI: 2.21, 22.11) and being moderately/very stressed (AOR: 3, 95% CI: 1.17, 7.89). Significant determinants of low tangible support were difficulty managing available income (AOR = 3, 95% CI: 1.29, 6.95), and being depressed (AOR = 2.8, 95% CI: 1.48, 5.34).

**Conclusion:**

The study revealed that 7.1% of pregnant women reported low social support. Having a mental health problems, being stressed, being from low socio-economic status and being non-partnered were significant determinants of low social support during pregnancy. Maternal health professionals and policymakers can use this information to screen pregnant women at risk of receiving low social support and improve the level of support being provided.

## Plain Language Summary

Providing strong social support improves emotional and physical well-being, strengthens social relationships, promotes health, and enhances the stress coping ability of pregnant women. However, pregnant women receiving low social support are at risk of substances use, developing mental illness, and adverse birth outcomes. The current study aims to determine the prevalence and determinants of low social support during pregnancy among Australian women. Data were obtained from Survey 6 of the 1973–1978 cohort of the Australian Longitudinal Study on Women’s Health (ALSWH) (n = 493, pregnant women aged 34–39 years). This cohort is one of ALSWH ongoing community-based study focusing on the health of women in Australia. Social support provided for a pregnant woman was the outcome variable, assessed using the 19-item Medical Outcomes Study Social Support index (MOSS). The prevalence of low social support in each domain was calculated for each of the independent variables. A logistic regression model, using a backward stepwise elimination approach, was employed to identify determinants of low social support. In the final model, the strength of association measured by adjusted odds ratios (AOR) with 95% confidence intervals. The significance level was set at p < 0.05. From the total participant pregnant women, 7.1% of them reported low social support. Significant determinants of low social support among pregnant Australian women were, having a mental illness, being stressed and/or being from low socio-economic status and being non-partnered. Maternal health professionals can use this information to screen pregnant women at risk of receiving low social support as well as develop policy to help enhance the social support being given and the psychological wellbeing being of pregnant women.

## Background

Pregnancy is an important but emotionally sensitive time for most women and it is also accompanied by changes in, physical appearance, role and lifestyles [1, 2]. Such changes may have an impact on the attitude, decision making and behaviour of the pregnant mother in undertaking the social responsibility that comes with pregnancy and motherhood [3]. These changes during pregnancy may be exacerbated by financial problems, relationship issues and lack of social support [4–6]. Thus, to tackle these challenges the need for social support during pregnancy is vital [7].

Social support is defined as the provision of emotional (e.g. caring), or informational (e.g. notifying someone of important information) support, instrumental (e.g. helping with housekeeping), tangible (e.g. practical support like financial aid), and/or psychological support for somebody by the social network of family members, friends, and community members [8]. It is an assistance from one individual or a social network that is given to another individual, which produces an instant or later positive response in the recipient [9]. Social support is assumed to influence through two potential mechanisms (i.e. main effects and buffering mechanism) [10–12]. As the main effect, social support and social network provide a sense of belonging and stability which result in improved self-esteem and reduce the risk of stress and mental illness [12]. Social support can act as a buffer by providing access to additional resources to enhance suitable coping mechanism for pregnant women to deal with stressful events [10, 12].

Providing strong social support improves emotional and physical well-being [13], strengthens social relationships, promotes health [14], and enhances the ability of pregnant women to cope with stress [15, 16]. Also, social support can reduce functional impairment among individuals with depressive symptoms, and increase the likelihood of recovery, thereby improving the overall quality of life (QOL) [17, 18]. It has been suggested that social support interventions and social participation are effective in preventing prenatal and neonatal adverse birth outcomes [19, 20]. Furthermore, social support can improve self-confidence, increase resistance to infections, and contribute to a healthier lifestyle [21, 22]. The vast bulk of research examining the relationship between social support and pregnancy outcomes over the past thirty years have shown individuals who receive good social support have greater longevity than those with poor social support [23].

Previous studies have shown that low social support is a risk factor for depression during pregnancy and post-birth [24–26], as well as a greater risk of giving birth to an underweight infant [27]. There appears to be a significant association between a lack of social support and drinking alcohol, smoking tobacco and illicit drugs use during pregnancy [28–30] and pregnant women who report anxiety disorder, who smoke tobacco, who live without a partner, who have an unplanned pregnancy and/or who have a low socio-economic status also report low social support [31]. Furthermore, a women’s level of education and length of relationship with their partner are also important determinants of social support during pregnancy [32].

Despite all this evidence, no study has addressed the prevalence and determinants of low social support during pregnancy among Australian women. Therefore, the purpose of the current study intended to fill this research gap by examining the prevalence and determinants of low social support during pregnancy amongst a sample of Australian pregnant women.

## Methods and materials

### Population, sampling and data collection

Data were obtained from the Australian Longitudinal Study on Women’s Health (ALSWH) [33, 34]. ALSWH was established in 1996 with the recruitment of 40,000 women in three different age cohorts (women born between 1973–1978, 1946–1951 and 1921–1926). The ALSWH project is designed to observe changes in women’s health through follow-up, provide information that will elucidate cause-and-effect relationships and examine the effects of changes in policy and practice.

ALSWH study participants were selected randomly (with over-sampling of women living in rural and remote areas, who were sampled at twice the rate of women in urban areas) using the national health insurance database (now Medicare Australia). Information from participants for the 1873–1878 cohort was gathered via mailed surveys on a three-year interval. The age of participating women of the 1973–1978 cohort was between 18 and 23 years when recruited in 1996 and were broadly representative of the population of similar age Australian women at that time [35]. Details about the ALSWH project have been published elsewhere [33, 34]. The sample for this study was drawn from Survey 6 of the 1973–1978 cohort. From the 8010 women who completed Survey 6 (aged 34–39 years), those who report being pregnant (n = 493) were included in the current analysis.

### Variables and measurement

Social support provided for a pregnant woman was the outcome variable, assessed using the Medical Outcomes Study Social Support index (MOSS) [36]. MOSS has an overall index of 19 items and 4 functional support subscales: emotional/informational support (involves caring, love and empathy) (8 items); tangible support (the provision of material aid or behavioral assistance) (4 items); and affectionate support/positive social interaction (involving expressions of love and affection/the availability of other persons to do fun things) (7 items). Each of the 19 items has a 5-point Likert response (ranging from: ‘none of the time’ = 1 to ‘all of the time’ = 5) assessing the availability of support. For the purpose of the current analysis, each domain of social support was categorized into high (“all of the time” and “most of the time”) and low (“a little of the time/none” and “some of the time”) social support [36].

Study participants were requested to specify their marital status as either “married,” “never married,” “de facto,” “separated,” “divorced,” or “widowed.” For the current analysis, groups were re-categorized into either “partnered” (married or de facto) or “non-partnered” (single, divorced, separated, or widowed). Postcode of residence was used to categorise respondents as living in either “major cities of Australia”, “inner regional Australia”, “outer regional Australia” or “remote or very remote Australia” [37]. Income stress was measured via how respondents reported ability to manage on available income, with response options: “impossible”, “difficult all of the time”, “difficult some of the time”, “not too bad”, or “easy”. For the purposes of analyses, these options were collapsed into 2 categories, “impossible or difficult all or some of the time” and “not too bad or easy”.

The respondents were questioned about their alcohol consumption. Based on their responses, the women were then categorised as being either a “non-drinker” or a “drinker” of alcohol. Similarly, based on their response to a question regarding cigarette smoking, the women were categorised as being either a “non-smoker” or a “current smoker”. To assess illicit drug use, women were provided with a list of drugs and asked if they had used any of them during the past 12 months. The list of drugs included Marijuana; Amphetamines; LSD; Hallucinogens; Tranquillizers; Cocaine; Ecstasy/designer drugs; Inhalants; Heroin; Barbiturates; and Steroids. Based on their responses, the women were classified as being either a “non-user” or a “user” of illicit drug.

The level of stress in the last 12 months among study participants was assessed using the Perceived Stress Questionnaire, which has been developed and validated for the ALSWH study [38]. The tool examined the level of perceived stress in specific areas of life, including study, relationships and own health. An overall mean stress score was determined, which ranges from 0 (no stress) to 4 (extreme stress). The Perceived Stress Questionnaire has good internal reliability (α = 0.75) [39, 40].

Depression was assessed using the 10-item Center for Epidemiological Studies Depression (CES-D-10) scale, with a possible range of 0–30, and Cronbach’s alpha 0.79. Cutoff point ≥ 10 from the total score indicates the presence of depressive symptoms [41]. CES-D-10 has been used to examine depressive symptoms during pregnancy with good reliability and validity [42–46]. The anxiety symptoms among pregnant mothers were assessed using the 9-item (yes/no) Anxiety subscale of the Goldberg Anxiety and Depression scale (GADS). A score of GADS greater than 6 indicates the presence of anxiety symptoms and has good reliability with Cronbach’s alpha of 0.77 [47].

### Data analysis

Data were analysed using SPSS version 22. Chi-square tests were used to test for crude associations between domains of social support and socio-demographic, mental health, and lifestyle behaviour related factors. Independent sample t-test analyses were performed to examine the difference in the mean age of pregnant women for each domain of social support. The prevalence of low social support in each domain was calculated for each of the independent variables. During the bivariate analyses variables with a p-value less than 0.25 entered into a multiple logistic regression model. In the final model, significant determinants of low social support were identified using a backward stepwise elimination approach. The strength of association measured by adjusted odds ratios (AOR) with 95% confidence intervals. The significance level was set at p < 0.05. The model fitness test was conducted using the Hosmer and Lemeshow goodness of fit test [48].

### Ethics approval and consent to participate

The ALSWH has been granted ethics clearance by the human research Ethics committee of the University of Newcastle (#H-076-0795) and the University of Queensland (#2004000224). Study participants were involved voluntarily and provided written informed consent. The confidentiality of study participants’ information is firmly monitored by ALSWH staff. Approval letter for the current study was obtained from the Human Research Ethics Committee of the University of Technology Sydney (ETH20-5306).

## Result

The mean age of participating women was 36.3 (standard deviation [SD] = 1.42). The majority of participants (78.5%) were married, while 37.6% attained a university degree. The majority of these women (58%) lived in major cities and 44.4% responded to having little or no difficulty in managing available income. The majority of the women (42%) were in the last trimester of their pregnancy, while 37.5% and 20.5% were in the second and first trimester respectively (Table 1). The overall prevalence of low social support among pregnant women was 7.1% (n = 35) (95% CI: 4.83, 9.39). Considering the specific domains of social support, the prevalence of low emotional support, low affectionate support and low tangible support in the current study was found to be 7.9%, 4.9%, and 10.9%, respectively. It was found that 25.1% of the women reported depressive and 20.9% reported anxiety symptoms.

**Table 1:** Demographic characteristics of participating pregnant women (n = 493)

Demographic characteristics	Number	Percent
Residence		
Major cities	286	61.1
Inner regional	103	22
Outer regional	66	14.1
Remote/very remote^a^	13	2.8
Occupation		
Manager/professional/Assoc. Professional	294	59.8
No paid job	100	20.4
Others^b^	97	19.8
Marital status		
Married/De facto (opposite sex)	466	94.8
Never married	12	2.4
Divorced/single/separated^c^	14	2.8
Highest qualification achieved		
University	319	65
Certificate/diploma	104	21.2
Year 12 or equivalent	43	8.8
Others^d^	25	5
Pregnancy month		
< 3 month	101	20.5
3–6 month	185	37.5
> 6 month	207	42
Able to manage on income available		
Difficult all the time/impossible^e^	43	8.8
Difficult some of the time	118	24
Not too bad	219	44.6
It is easy	111	22.6

^a^Remote (n = 6), Very remote (n = 7)

^b^Others (Tradesperson or related worker (n = 7), Advanced clerical or service worker (n = 33), Intermediate clerical, sales/service worker (n = 38), Intermediate production or transport worker (n = 1), Elementary clerical, sales or service worker (n = 11), Labourer or related worker (n = 7))

^c^Divorced (6), Separated (6), De facto (n = 2)

^d^Others (no formal education (n = 2), Year 10 or equivalent (n = 15), Trade/apprenticeship (n = 8))

^e^Impossible (n = 8), Difficult all the time (n = 35)

The bivariate associations between demographic factors and domains of social support during pregnancy are shown in Table 2. Women with low emotional support were more likely to be non-partnered (p < 0.001) and report that their ability to manage on available income was impossible/difficult all of the time (p < 0.001). Similarly, women with low affectionate support were more likely to be non-partnered and found it difficult all of the time/impossible to manage on their available income (p < 0.001). Also, women with low tangible support were more likely to find it difficult all of the time/impossible to manage on their available income (p < 0.001) and were more likely to be non-partnered (p = 0.002). There was a significant mean age difference between participants with low/high emotional support (p = 0.021), and low/high affectionate support (p = 0.021).

**Table 2:** Demographic factors associated with low social support domains during pregnancy among Australian women (n = 493)

Demographic characteristics	Emotional support		p-value	Affectionate support		p-value	Tangible support		p-value
	Low (n = 39)	High (n = 453)		Low (n = 24)	High (n = 467)		Low (n = 52)	High (n = 439)	
	n (%)	n (%)		n (%)	n (%)		n (%)	n (%)	
Residence									
Major cities	17 (43.5)	269 (59.4)	0.093	11 (45.8)	274 (58.6)	0.399	27 (52)	259 (59)	0.42
Inner regional	12 (30.7)	90 (19.8)		7 (29.2)	95 (20.4)		14 (27)	88 (20)	
Outer regional/remote/very remote	9 (23.07)	70 (15.4)		5 (25)	74 (16)		10 (19)	68 (16)	
Marital status									
Partnered	32 (82)	436 (96.2)	< 0.001	20 (83.3)	447 (96)	0.006	45 (86.5)	422 (96)	0.002
Non-partnered	7 (18)	17 (3.8)		4 (16.7)	20 (4)		7 (13.5)	17 (4)	
Highest qualification achieved									
University	20 (51.3)	299 (66)	0.065	13 (54.2)	305 (65.5)	0.209	33 (63.5	285 (65)	0.21
Certificate/diploma or trade/apprenticeship	10 (25.6)	102 (22.5)		9 (37.5)	103 (22.1)		9 (17.3)	103 (23.6)	
School only	9 (23.1)	51 (11.5)		2 (8.3)	58 (12.4)		10 (19.2)	50 (11.4)	
Pregnancy months									
< 3 month	6 (15.4)	95 (20.9)	0.62	4 (16.7)	97 (20.7)	0.96	8 (15.4)	93 (21.2)	0.246
3–6 month	17 (43.6)	168 (37)		11 (45.8)	174 (37.3)		25 (48.1)	160 (36.4)	
> 6 month	16 (41)	190 (41.9)		9 (37.5)	196 (42)		19 (36.5)	186 (42.4)	
Able to manage on income available									
Impossible/difficult all of the time	13 (33.3)	30 (6.6)	< 0.001	8 (33.3)	35 (7.5)	< 0.001	13 (25)	30 (6.8)	< 0.001
Difficult some of the time	9 (23.1)	109 (24.1)		3 (12.5)	115 (24.6)		14 (26.9)	103 (23.5)	
It is easy/not bad	17 (43.6)	313 (69.2)		13 (54.2)	317 (67.9)		25 (48.1)	305 (69.6)	

	Mean (SD)	Mean (SD)	p-value	Mean (SD)	Mean (SD)	p-value	Mean (SD)	Mean (SD)	p-value
Age	36.78 (1.5)	36.3 (1.40)	0.035	36.9 (1.42)	36.28 (1.4)	0.021	36.5 (1.44)	36.3 (1.41)	0.234

p-value was based on chi-square test and t-test statistics

Table 3 shows the bivariate associations between mental health and lifestyle behaviour related factors and domains of social support. Women with low emotional support more likely to report being depressed (p < 0.001), anxiety (p < 0.001), diagnosed or treated for depression in the past 3 years (p < 0.001), diagnosed or treated for anxiety in the past 3 years (p = 0.021) and being moderately/very stressed (p < 0.001) during pregnancy compared with participants getting high emotional support. Similarly, women with low affectionate support were more likely to report being depressed (p < 0.001), anxiety (p < 0.001), diagnosed or treated for anxiety in the past 3 years (p = 0.042) and being moderately/very stressed (p < 0.001). In addition, women with low tangible support were more likely to report being depressed (p < 0.001), anxiety (p = 0.001), diagnosed or treated for depression in the past 3 years (p = 0.002), diagnosed or treated for anxiety in the past 3 years (p = 0.007) and being moderately/very stressed (p = 0.002).

**Table 3:** Mental health and lifestyle behaviour related factors associated with low social support during pregnancy among Australian women (n = 493)

Variables	Emotional support		p-value	Affectionate support		p-value	Tangible support		p-value
	Low (n = 39)	High (n = 453)		Low (n = 24)	High (n = 467)		Low (n = 52)	High (n = 439)	
	n (%)	n (%)		n (%)	n (%)		n (%)	n (%)	
Alcohol use									
Non-drinker	15 (38.5)	132 (29.1)	0.185	11 (45.8)	135 (28.9)	0.079	17 (32.6)	130 (29.6)	0.59
Low/High risk drinker	23 (60)	320 (70.6)		13 (54.2)	330 (70.6)		34 (65.4)	308 (70.2)	
Tobacco smoking									
Current smoker	2 (5.1)	20 (44.2)	0.836	1 (4.2)	21 (4.5)	0.930	4 (7.7)	18 (4.1)	0.236
Non-smoker	37 (94.9)	433 (95.6)		23 (95.8)	446 (95.5)		48 (92.3)	421 (95.9)	
Illicit drug use									
Yes	27 (69.2)	291 (64.2)	0.531	17 (70.8)	300 (64.2)	0.510	37 (71.2)	281 (64)	0.308
No	12 (30.8)	162 (35.8)		7 (29.2)	167 (35.8)		15 (28.8)	158 (36)	
Depressive symptoms (CES-D 10)									
< 10	7 (17.9)	358 (79)	< 0.001	4 (16.7)	361 (77.3)	< 0.001	24 (46.2)	341 (77.7)	< 0.001
≥ 10	32 (82.1)	90 (21)		20 (83.3)	101 (21.7)		27 (51.2)	95 (21.6)	
Depression in the past 3 years ( Previous Mental health)									
Yes	13 (33.3)	45 (10.1)	< 0.001	5 (20.8)	53 (11.5)	0.172	13 (25)	45 (10.4)	0.002
No	26 (66.7)	400 (89.9)		19 (71.2)	406 (88.5)		39 (75)	386 (89.6)	
Anxiety symptoms (GAD)									
< 6	15 (38.5)	374 (82.6)	< 0.001	5 (20.8)	384 (82.2)	< 0.001	32 (61.5)	356 (81.1)	0.001
≥ 6	24 (61.5)	79 (17.4)		19 (79.2)	83 (17.8)		20 (38.5)	83 (18.9)	
Anxiety in the past 3 years ( Previous Mental health)									
Yes	6 (15.4)	26 (5.8)	0.021	4 (16.7)	28 (6.1)	0.042	8 (15.4)	24 (5.6)	0.007
No	33 (84.6)	419 (94.2)		20 (83.3)	431 (93.9)		44 (84.6)	407 (94.4)	
Stress									
Not at all/somewhat stressed	22 (56.4)	396 (87.4)	< 0.001	10 (41.7)	407 (87.2)	< 0.001	37 (71.2)	380 (86.6)	0.002
Moderately/very stressed	17 (43.6)	55 (12.1)		14 (58.3)	58 (12.8)		15 (28.8)	57 (13.4)	

p-value was based on chi-square test statistics

The significant factors associated with low social support during pregnancy are presented in Table 4. After adjusting for confounding variables, multiple logistic regression found that non-partnered pregnant women (AOR = 4.4; 95% CI: 1.27, 14.99; p = 0.019), difficulty all the time/impossible to manage on available income (AOR = 3.1; 95% CI: 1.18, 8.32; p = 0.023), suffering from depressive symptoms (AOR = 8.5, 95% CI: 3.29, 22.27, p < 0.001) and having anxiety symptoms (AOR = 2.9; 95% CI: 1.26, 7.03; p = 0.013) were more likely to receive low emotional support. Pregnant women with depressive symptoms (AOR = 5.3; 95% CI: 1.59, 17.99; p = 0.007), anxiety symptoms (AOR: 6.9; 95% CI: 2.21, 22.11; p = 0.001) and moderately/very stressed (AOR: 3; 95% CI: 1.17, 7.89; p = 0.023) were more likely to receive low affectionate support compared with their counterpart. Further, pregnant women who find it difficult all the time/impossible to manage on their available income (AOR = 3; 95% CI: 1.29, 6.95; p = 0.010) and those with depressive symptoms (AOR = 2.8; 95% CI: 1.48, 5.34; p = 0.002) are around three times more likely to receive low tangible support.

**Table 4:** Determinants of domains of social support during pregnancy among Australian women (n = 493), as determined by backward stepwise elimination approach using Multiple logistic regression modelling

Variables	Emotional support		Affectionate support		Tangible support	
	AOR (95% CI)	p-value	AOR (95% CI)	p-value	AOR (95% CI)	p-value
Marital status						
Partnered	1.00		1.00		1.00	
Non-partnered	4.4 (1.27, 14.99)	0.019	4.1 (1.004, 17.15)	0.053	2.5 (0.90, 7.08)	0.077
Able to manage on income available						
Impossible/difficult all of the time	3.1 (1.18, 8.32)	0.023	–	–	3 (1.29, 6.95)	0.010
Difficult some of the time	1.2 (0.48, 3.11)	0.669	–	–	1.3 (0.63, 2.72)	0.479
It is easy/not too bad	1.00				1.00	
Depressive symptoms (CES-D 10)						
< 10	1.00		1.00		1.00	
≥ 10	8.5 (3.29, 22.27)	< 0.001	5.3 (1.59, 17.99)	0.007	2.8 (1.48, 5.34)	0.002
Anxiety symptoms (GAD)						
< 6	1.00		1.00		–	
≥ 6	2.9 (1.26, 7.03)	0.013	6.9 (2.21, 22.11)	0.001	–	–
Depression in the past 3 years (previous Mental health)						
Yes		–	–	–	2 (0.94, 4.34)	0.073
No		–	–		1	
Stress						
Not at all/somewhat stressed	–		1.00		–	
Moderately/very stressed	–	–	3 (1.17, 7.89)	0.023	–	–

_For low emotional support: variables entered in step 1; Residence, Marital status, highest qualification, able to manage on income available, current depressive symptoms (CESD- 10), current anxiety symptoms (GAD), Depression in the past 3 years, Anxiety in the past 3 years, Stress. P-value of Hosmer and Lemeshow test=0.939_

_For low affectionate support: variables entered in step 1; Marital status, highest qualification, able to manage on income available, alcohol use, current depressive symptoms (CESD- 10), current anxiety symptoms (GAD), Depression in the past 3 years, Anxiety in the past 3 years, Stress. P-value of Hosmer and Lemeshow test=0.287_

_For low tangible support: variables entered in step 1; Marital status, highest qualification, able to manage on income available, pregnancy months, Tobacco smoking, current depressive symptoms (CESD- 10), current anxiety symptoms (GAD), Depression in the past 3 years, Anxiety in the past 3 years, Stress. P-value of Hosmer and Lemeshow test=0.965_

_*CI* Confidence interval, *AOR* Adjusted Odds Ratio, *CES-D 10* Center for Epidemiological Studies Depression scale, *GAD* Goldberg Anxiety and Depression scale_

## Discussion

This study is the first to determine the magnitude and determinants of low social support during pregnancy among Australian women. The overall prevalence of low social support among pregnant women was 7.1%. The primary risk factors identified across the domains of low social support (i.e. low emotional, low affectionate and low tangible supports) were pregnant women’s marital status, ability to manage available income, and mental health issues.

The current study found that those pregnant women who were not partnered were 4.4 times more likely to receive low emotional support, compared with partnered pregnant women. Marital Partner is one of the important sources of emotional and affectionate support [49] and support from spouse and marital stability are an important protective factor for the mental well-being of women during pregnancy [50]. Individual studies revealed that lack of support from a companion was more common among those who were not partnered [51, 52]. Also, compared with non-partnered women, partnered women generally have numerous psychological and social advantages, though much of this may be limited to individuals living in a satisfactory marital relationship [53] because pregnant women living in a violent relationship with their partner are more likely to receive less social support [54]. Our finding is supported by previous studies conducted in US [55], Sweden [51] and Portugal [52]. However, cross-sectional study conducted in Iran (n = 320) [56] and Southern Brazil (n = 871) [31] among a sample of pregnant women reported that marital status has no significant association with social support. The possible reason for this inconsistency might be due to the difference in the social support tool used, the demographic characteristics of participants, and the variation in the understanding of social support across individual participant because of the difference in socio-economic settings and cultural variation across countries. For instance, the study conducted in Iran includes participants with age ≥ 18 years (mean age 25.7 ± 5.5 years) and used a 23 item Vaux Social Support Questionnaire with three domains (support from family, friends and relatives), whereas our study includes participants with the age range of 34 to 39 years (mean age 36.3 ± 1.42 years) and used 19 item MOSS scale which examined the emotional/informational support, affectionate support/positive social interaction and tangible support. Additionally, the non-significance of marital status in a study conducted in Iran might be due to small number of non-partnered pregnant women (n = 1) which can limit the power of the analysis.

Our study shows that pregnant women who have difficulty in managing available income are three times more likely to receive low emotional and/or low tangible support compared to those women who receive high emotional and/or high tangible support. This concept was supported by a study conducted in Germany, which identified that socially disadvantaged persons more often report poor social networks and social support compared with their counterpart [57]. Also, pregnant women with a higher household income predict better perceived social support based on reports of a study conducted in Mexico [58]. It is argued that individuals living in low socio-economical class tend to have a more limited relational radius and prefer self-isolation from the social events because involvement in social activities needs money to afford events which might lead them to self-neglect from the society [59]. Moreover, withdrawal from society and having less friends may lead them to the feeling of loneliness, receive low emotional support and/or low affectionate support from the social environment including family, friends and spouse [60].

Our study identifies depression during pregnancy as positively associated with the three domains of social support (i.e. low emotional/informational support, low affectionate/positive social interaction and low tangible support). The possible reason could be, individuals with depression tend to withdraw themselves from social support and thus communicate with fewer family member/friends or they might feel more mistrustful and underestimate the level of existing support [61]. Also, in the current study, participants were asked to report if they experienced depressive symptoms in the past week before the day of filling the questionnaire. Therefore, past week depressive symptoms could be strongly associated with low social support because being depressed may affect the perception of social support which might lead to underestimating the support received [62]. Besides, depressed individuals induce negative responses and create interpersonal difficulties in their interactions with others, which can lead to avoidance or rejection by others [61, 63, 64]. The interpersonal accounts of depression also suggest that negative self-evaluation and social inadequacy manifested by depressed individuals disrupt social interactions [65, 66] and as a result, an individual could be at risk of receiving low social support from family, friends and community. The finding in the current study supported by a cross-sectional study conducted in Iran among pregnant women (n = 320), which revealed a significant inverse relationship between depression and total social support score (p < 0.001), family support (p < 0.001), friends support (< 0.001) and relative’s support (p = 0.003) [56].

Our study shows pregnant women who have anxiety symptoms are almost three and nine times more likely to receive low emotional and low affectionate support respectively, as compared to those without anxiety symptoms. It is known that anxiety impairs social interaction [31], which explains why individuals with this disorder tend to be lonely and did not have the need to communicate. However, it is also possible that individuals who have anxiety symptoms are mainly involved by their own concerns and did not acknowledge the support received [67]. The finding of our study supported by, a cross-sectional study conducted in Southern Brazil (n = 871) which found a moderate association between anxiety and affectionate, emotional, tangible, and informational domains; and a strong association with the positive social interaction domain [31]. Additional support for our findings comes from, another cross-sectional study conducted in Iran among pregnant women (n = 320) which found a significant inverse relationship between antenatal anxiety and total social support score (p < 0.001), family support (p < 0.001), and friends support (< 0.001) [56].

Being moderately/very stressed during pregnancy is significantly associated with low affectionate support, as shown in our study. The possible reason for this might be, people with high levels of stress may be less able to keep contact and form a strong social relationship with other people; thus, keeping others in distance probably because they have a fear that they might convey their high stress to their social networks [68]. It is also possible that people with high levels of perceived stress underestimate the social support they receive and did not use support in a way that could benefit their psychological well-being [69].

Some limitations need to be considered when interpreting our study findings. First, the findings rely on self-reported data from study participants and therefore are prone to recall bias. Second, our findings are limited to pregnant women within the age range of 34–39 years (i.e. younger pregnant women not included) and this was because of undertaking a secondary analysis of a preexisting database. Third, our study didn't assess the social class of study participants, as a result, we couldn't stratify the marital status of study participants with their corresponding social class to see if it has an effect on marital status in predicting domains of social support. Fourth, the use of a cross-sectional study design in the study raises concerns about reverse causation between low social support and depressive and/or anxiety symptoms. The cross-sectional study design cannot ensure the necessary time-based order of events. Specifically, if low social support (i.e. the exposure variable) precedes, it may cause individuals to develop depressive and/or anxiety symptoms (i.e. the outcome variable). In contrast, the early presence of depressive and/or anxiety symptoms may cause an individual to receive low social support. Therefore, reverse causation is possible, which creates a vicious circle that cannot be accurately modelled in a cross-sectional design. However, analyzing data collected from a nationally representative sample of pregnant women is the strength of our study.

Overall, the current study offers evidence of sociodemographic, psychosocial and lifestyle behavioural determinants of low social support, and a better understanding of these risk factors will allow for more targeted screening to identify pregnant women who are at risk of receiving low social support. Further research identifying the experience and details of additional risk factors for low social support amongst pregnant women is needed to help inform intervention strategies to improve the physical and psychological well-being of Australian pregnant women. Finally, to address the issue of reverse causation in the associations between low social support and depressive and/or anxiety symptoms, future longitudinal studies, which can ensure the temporal order of event, particularly over the different stages of pregnancy is strongly recommended.

## Conclusions

Seven Percent of pregnant women in Australia reported low social support during their pregnancy. Having a mental health problems (depressive and/or anxiety symptoms), experiencing stress, being at a low socio-economic status, and not being in a marital relationship are all significant determinants of low social support. Maternal health professionals can use this information to screen pregnant women at risk of receiving low social support as well as develop policy to help enhance the social support being given and the psychological wellbeing being of pregnant women.

## Data Availability

After request, all analyzed data will be available from the Australian Longitudinal Study on Women’s Health (ALSWH). https://www.alswh.org.au/

## Abbreviations

ALSWH: Australian Longitudinal Study on Women’s Health
AOR: Adjusted odds ratio
BAI: Beck Anxiety Inventory
CES-D-10: 10 Item Center for Epidemiological Study Depression scale
CI: Confidence interval
C/S: Cesarean section
DUSOCS: Duke Social Support and Stress Scale
EPDS: Edinburgh Postnatal Depression Scale
GADS: Goldberg Anxiety Depression Scale
GP: General practitioner
LMICs: Low and middle income countries
LOT-R: Life Orientation Test Revised
MOS-SSS-19: 19 Item Medical Outcome Study Social Support Scale
STAI: State-Trait Anxiety Inventory
WHO: World Health Organization
