# Correction to: Prevalence and determinants of low social support during pregnancy among Australian women: a community-based cross-sectional study

**DOI:** 10.1186/s12978-021-01231-7

**Published:** 2021-09-21

**Authors:** Asres Bedaso, Jon Adams, Wenbo Peng, David Sibbritt

**Affiliations:** 1 College of Medicine and Health Sciences, Faculty of Health, School of Nursing, Hawassa University, Hawassa, Ethiopia; 2 Australian Centre for Public and Population Health Research, School of Public Health, Faculty of Health, University of Technology Sydney, Ultimo, NSW Australia

## Correction to: Reprod Health (2021) 18:158 https://doi.org/10.1186/s12978-021-01210-y

Following publication of the original article [1], the authors reported some variation between the descriptive section and the percentages presented in Table 1 for some variables.

The original article [1] has been updated.
